# Theoretical perspectives: sociology and the conservation of scientific heritage

**DOI:** 10.3389/fsoc.2024.1473206

**Published:** 2024-11-01

**Authors:** Stefano Magnolo, Ana Galán-Pérez

**Affiliations:** ^1^Department of Legal Studies, University of Salento, Lecce, Italy; ^2^Department of Painting and Conservation-Restoration, Complutense University of Madrid, Madrid, Spain

**Keywords:** scientific heritage, critical conservation, digital humanities and heritage, sociology, social systems theory

## Abstract

The following considerations adopt a critical conservation approach to understanding scientific heritage, particularly its intangible aspects. This heritage includes the intellectual and research legacy, encompassing various forms of communication, with a focus on digital technologies. Conservation methods now play a crucial role in transmitting this intangible heritage, shifting from traditional substance-based care to communication systems and enhancement facilitated by digital humanities. These advancements enable novel experiences and foster new academic and social practices. Interestingly, sociology features prominently in this context. It appears twice: first as a discipline that produces intangible heritage worthy of preservation and communication, and second as a theory for communicating this heritage. Our exploration begins by recognizing the university’s role as a cultural agent engaged in producing and transmitting knowledge. We then delve into the concept of scientific heritage, particularly how the Humanities and Social Sciences preserve their heritage compared to the so-called hard sciences. While we acknowledge the importance of the real impact of these disciplines, our focus is on the formal recognition of their scientific production as cultural heritage. Ultimately, we focus on specific heritage worth preserving and reflect on ways to enhance it in the future.

## Introduction

The university plays a crucial role as a cultural agent. If the progress achieved within universities or research organizations is not effectively shared with society, the entire endeavor of scientific research loses its meaning.^1^

We recognize that the prevailing university model, often referred to as the Humboldtian model, revolves around two fundamental tasks: teaching and research. In recent years, these two tasks have been joined by what is called the third mission, which concerns social engagement with communities and territories (Compagnucci and Spigarelli, 2020). Through this teaching-research-community engagement synergy, universities become agents of change, influencing minds by transmitting knowledge they themselves help create. Simultaneously, they foster critical thinking skills necessary to engage with societal challenges.

Within this context, it is essential to distinguish between what is formal and what is real. The formal aspect refers to the tangible outputs of academic work, such as publications, presentations, and awards. These are the visible markers of scholarly activity. On the other hand, the real aspect pertains to the actual impact of this work on society, including policy changes, educational reforms, and societal advancements. While our focus is on the formal recognition of the scientific production in the Humanities and Social Sciences as cultural heritage, we also draw attention to the scientific value of research in these disciplines as opposed to the hard sciences, which appear to be more accredited. Moreover, it is precisely the real impact that our disciplines have on modern societies^2^ that constitutes part of their intangible value, i.e., that which transcends the formal data but is conveyed by the “good” on which we focus with respect to its preservation and proper communication. To this end, questions related to heritage conservation decisions are approached critically and individually. As both members of the university community and active citizens, we ponder how to identify and preserve culturally significant heritage.

The transfer of knowledge in the Humanities and Social Sciences, considering the role of new technologies, is fostered by Education and Teaching Innovation projects. These initiatives allow for the application of novel teaching methods in cultural heritage conservation, particularly within the framework of digital humanities. By training future researchers, we facilitate knowledge dissemination. As these researchers contribute to new knowledge, their work gains social recognition and becomes part of our cultural heritage. Unfortunately, this humanistic transfer seems less prevalent in non-academic settings, where communication tends to focus on the so-called hard sciences. Consequently, our rich academic heritage often remains overlooked and unacknowledged (Ebers, 2022, pp. 55–75).

To identify risk factors for the loss of scientific heritage in the Humanities and Social Sciences and explore preservation strategies, research groups are being mobilized.^3^ Within this research framework, the goal is to recognize the results of research activity as an intangible heritage that contributes to cultural enrichment and generates new knowledge. Additionally, these efforts produce tools for learning and teaching.^4^

Inspired by the theory of critical heritage conservation, innovative educational projects emerge from a dialog involving all stakeholders. Through this dialog, both tangible and intangible heritage are identified, acknowledged, and preserved. This preservation mechanism aims to positively impact present and future cultural experiences, fostering a common good within and beyond the university.

Considering that universities safeguard a rich heritage resulting from their research and teaching dynamics, we are exploring how to assess the value of scientific production and ensure its preservation and transmission. As a reference point, we look to the protection of scientific collections within university museums.

### State of the art: the concept of scientific heritage

According to Lourenço and Wilson (2013), ‘scientific heritage is diverse, complex, multifaceted, and more difficult to define than industrial or natural heritage.’ This complexity arises not only from its inclusion of all heritage resulting from scientific development but also from its encompassing sites, landscapes, research buildings, technological instruments, and natural science samples.

Defining scientific heritage is challenging, and integrating immaterial aspects into a comprehensive definition poses even greater difficulty. Academic and professional studies have traditionally prioritized the preservation of tangible university collections. Consequently, we must consider how to identify and preserve the immaterial heritage arising from the scientific process, enhancing its value.

Within the framework of digital humanities, semantic searches—emphasizing language, keywords, and metadata—have been conducted. These searches reveal that the term ‘scientific heritage’ serves as a synonym for researchers’ scientific production.^5^

#### Scientific heritage and hard sciences

In the hard sciences, numerous essays recognize researchers’ biographies and their scientific production as scientific heritage. For instance, Aron Gutman’s work is described as a catalog of research areas in medicine (Baginskas et al., 2009). Similarly, the biography and research of Soviet mathematician Boris Lukich Laptev are documented (Bliznikas et al., 1991), as are those of Romanian physicist Alexandru Proca (Calboreanu, 2004). Professor Nikoloz Beruchashvili’s scientific legacy in geography and landscape sciences transcends various branches of geography and cartography (Gachechiladze et al., 2017).

At the University of Bucharest, a session commemorating the centenary of the invention of the first jet aircraft highlights the current impact of Henri Coanda’s scientific heritage. Coanda’s work introduced novel ideas to data processing, project management, and social and technological sciences in aviation (Haret et al., 2010). In the field of biology, we also observe the equation of scientific heritage with researchers’ biographies and academic development. Tatyana Batygina, a researcher and professor at St. Petersburg State University, exemplifies this (Titova et al., 2018).

Furthermore, an article by authors from Pavlodar University and Nur-Sultan University of Kazakhstan delves into the life and scientific legacy of A.ZH. Mashanov, a Doctor of Mineralogy and Geology. Mashanov’s work extends beyond geology, encompassing archaeology, history, significance, theology, and Farabi studies. The article reflects both his impact on culture and the challenges of conducting research in the 1960s. The authors conclude with the powerful statement: ‘The scientist’s legacy clearly reflects the cultural values of the Turkish people; his work teaches us to be proud of our people’s history and to preserve our national spirit.’ This cultural and identity aspect linked to his scientific legacy is indeed intriguing to analyze (Shabambayeva and Abdrahmanov, 2020).

The field of historians of science and the places associated with researchers are equally intriguing. In an academic review on scientific heritage published in the journal “Studies in History and Philosophy of Science,” the author discusses the segmented and micro-fragmented activities of science historians, as well as the relationship between scientific heritage and science museums (Fara, 1997). In another article, the same author explores Isaac Newton’s scientific legacy and the locations where he pursued his academic career—referred to as “sites of memory.” Notably, the commemoration of Newton’s memory at these sites not only reinforces his status as a scientific genius but also promotes various interpretations of scientific and national heritage (Fara, 2000).

The search conducted using the keywords “scientific heritage” has revealed a broader context beyond mere preservation. It encompasses sites of memory, biographical details, researcher identities, and their cultural backgrounds.

Another fascinating aspect emerges from the methodology: the geographical origins of the researchers cited in this paper, primarily from the Eurasian continent. This prompts us to consider external factors that may have influenced the construction of this meaningful legacy among researchers with scientific heritage.

#### Scientific heritage and soft sciences

What if we apply the same terms to a search for scientific production in the soft sciences? The equation of scientific heritage with scientific production extends beyond natural sciences and technology. For instance:

- The study of literary scholar Romen Gafanovitch Nazirov’s contribution to the so-called scientific heritage (Borisova, 2014).
- Researcher E.G. Pchelina’s scientific heritage in the field of archaeology and ethnography, emphasizing interdisciplinary research. However, most of his manuscripts remain inaccessible for scientific study (Chibirov, 2016).
- An analysis of research in social anthropology in Bulgaria, where the title “scientific heritage” is synonymous with scientific activity and production (Draganov, 1992).

Additionally:

- Author L. Grebnev emphasizes the scientific heritage of Karl Marx’s work, continuing debates on “Marxism: between scientific theory and ‘secular religion’ (liberal apology)” (Grebnev, 2004).
- Researcher Shakirova (2017) discusses Babadjan Sanoi’s scientific heritage related to Sufism and the philosophical aspects of human beings portrayed through artistic characters. The essay explores how scientific and religious heritage influence the researcher’s production. It spans literature, philosophy, and religion, asserting that Sufism’s theory and practice constitute a universal religious heritage.

It is also interesting to use the term “epistolary heritage” to introduce the scientific enrichment resulting from the relationship between research professors Vitaly Epifanovich Larichev, Alexei Pavlovich Okladnikov, and Andrey Fedorovich Palashenkov. Through the exchange of unpublished letters among these three Siberian researchers, we gain insights into developments in historical sciences. These letters reveal discoveries related to Palaeolithic, Neolithic, Bronze, and Iron Age cultures, as well as advancements in archaeology, history, and ethnography (Мakhat, 2021).

The concept of scientific heritage extends beyond tangible artifacts. For instance:

- At Kazan Federal University, researchers apply methodological heritage to the teaching of the Russian language. Their approach addresses linguistic challenges by considering both the textual organization of teaching materials based on comparative typological analysis of Russian and native languages, and holistic education that fosters dialog between languages and cultures. Additionally, they emphasize a competence-based approach to developing bilingual linguistic identity (Fazliakhmetov and Yusupova, 2018).
- In the context of universities, the *Revista pedagógica de la Universidad de Cienfuegos* (Cuba) delves into heritage as a symbolic asset linked to identity. University identity emerges from various manifestations and social impact. Beyond its historical connections and professional training, university identity encompasses tangible and intangible aspects inherent to the institution. Moreover, in terms of sustainable development, the article (Fonseca Martínez and Brull González, 2020) emphasizes the human heritage cultivated within universities—a heritage that should benefit highly qualified citizens. This intriguing narrative recognizes that university heritage encompasses everything that shapes its identity, emphasizing its intangible nature, and explores methods for preserving this vulnerable heritage.
- In the realm of intellectual thought, few attempts in the literature consider it as intangible heritage. Notably, some researchers explore the concept of philosophical heritage. Although often associated with the place of production and interpreted through the lens of cultural tourism, this perspective transcends the limitation of scientific discourse to the “hard” sciences. According to these authors, “philosophy is the immaterial heritage of humanity, relevant in its totality and interdependent with its material heritage—its written works and spaces of reflection” (Sánchez Cotta, 2022).
- Expanding the context, we encounter elements such as epistolary heritage, identity, methodological heritage, advocacy, and social and political engagement. These enrich the discussion beyond the scientific heritage associated with the hard sciences.

In attempting to define intangible scientific heritage, we can conclude that in both fields of science there are elements that are identifiable and worthy of protection. But how to go about it?

### A paradigm shift (1): from scientific heritage as technological heritage to scientific heritage as intangible heritage

In attempting to define intangible scientific heritage, we can conclude that both fields of science contain identifiable and worthy elements for protection.

However, there is a paradigm shift from considering scientific heritage solely as technological (material) heritage (Rosell, 2000) to recognizing it as intangible heritage. Science generates a wide variety of material and immaterial heritage through literature, philosophy, history, art, anthropology, sociology, jurisprudence, and other disciplines. In this new integrative perspective, we refer to it as Research Heritage—a broader notion than the one used to define Scientific Heritage (Arroyo Serrano, 2022).

From the scientific community’s viewpoint, research heritage represents its identity and is worth passing on to the next generation of scientists and society as a whole. It encompasses everything we know about life, nature, and the universe, as well as how we acquire that knowledge. Its vehicle is both material and immaterial (Lourenço and Wilson, 2013).

Within this dual nature of scientific heritage, we find examples of protecting its vehicles of knowledge, such as photographic collections or old reels of slides in the context of history or art history education. Additionally, notebooks and travel diaries with manuscripts and drawings play a significant role for researchers, emphasizing the importance of transmitting both material and immaterial aspects.

The paradigm shift from considering scientific heritage solely as technological (material and technical) to recognizing it as immaterial heritage can be supported by the definition of heritage in the 2014 UNESCO Culture for Development Indicators: ‘Cultural heritage, in its broadest sense, is both a product and a process that provides societies with a wealth of resources…’ (UNESCO, 2014). In other words, the ‘act,’ the ‘experience,’ and the heritage itself serve as pathways to knowledge. We can integrate teaching methods, habits, rituals, and communication dynamics between scholars and students or among scholars. Even the relationship with academic spaces contributes to accessing the heritage or the scientific work generated by research (Stichweh, 2006).

On the other hand, as discussed in the first part of this paper, there are differences among scientific disciplines regarding how research results are communicated in the academic environment (Longo and Magnolo, 2009, 829). However, we can observe that the immaterial values identified in both hard and soft sciences are sometimes mutable and shared (such as biography, didactics, and places of memory). The boundaries are not always clear, and there exists a certain dynamism in communication and transmission. Visualizing these connections as connecting nodes, aided by digital humanities and their data visualization tools (similar to how UNESCO presents links between intangible cultural heritage), can enhance our understanding.^6^

### A paradigm shift (2): moving toward critical conservation

After identifying the elements that constitute this intangible heritage, the subsequent step involves establishing techniques for conserving it, which presents the added difficulty of preserving the intangible.

Over the past 15 years, a key challenge in academic research has been examining scientific heritage from the perspectives of museology and conservation to safeguard and enhance it (Soubiran, 2008; Boudia and Soubiran, 2013; Lourenço and Wilson, 2013). Taxonomies of academic legacy and formulas for its custody have been deeply explored and debated.^7^ The study primarily revolves around two museum networks that include university institutions and collections. On one hand, there is Universeum, the European Academic Heritage Network founded in 2010, where the aforementioned authors share their studies. On the other hand, there is the ICOM-UMAC network, the International Committee for University Museums and Collections, created in 2001, which has yielded findings consistent with those of the aforementioned network. ICOM-UMAC’s main objective is to promote interdisciplinary and creative usage of academic heritage in higher education. Their primary goal is to impart both tangible and intangible culture associated with higher education institutions to forthcoming generations. This commitment considers comprehensively addressing significant contemporary challenges and controversies (Lourenço and Wilson, 2013, p. 6).

Conserving university collections presents challenges due to the concept of material and immaterial duality. From the perspective of preserving cultural property, the significance of materiality—whether organic or inorganic—cannot be denied. Identifying the causes of deterioration and their pathologies, with the aim of halting or mitigating their effects, forms the core of theoretical and ethical reflections in strategic conservation, applied experimental sciences, and heritage management (Piñeiro-Naval and Frutos Esteban, 2023). However, in practice, the distinction between tangible and intangible cultural heritage is not always straightforward. Moreover, intervening in the physical components and values of cultural heritage may modify its significance.

This ongoing discussion within the field of conservation over the past two decades provides a solid foundation for developing a critical approach to conservation understood as a communication activity within the broader framework of a sociological theory of communication and society. It allows us to examine our professional identity and recognize that our work preserves not only tangible but also intangible values.

The identification and preservation of intangible heritage emerge within the context of a new scientific culture aimed at the general public, responding to the ongoing debate between science and society. Authors like Soubiran emphasize that only through commemoration and communication from universities can we witness the transition from academia to communities (Soubiran, 2008). Therefore, preserving this legacy holds great interest for both institutions and the scientific community. Heritage serves as a tool for mediation, achieving various objectives such as reinforcing identity, discipline, and integrating knowledge into the cultural and social environment.

### The question of the material and immaterial nature of the scientific heritage

It is worth considering the duality of material and immaterial scientific expressions within this framework. This could manifest as a concrete medium, either analogue or digital, acting as a carrier, or as a system of intangible symbolic actions and representations that do not require such a medium (such as the oral transmission of scientific production), or as a combination of the two. Recognizing a definition for intangible scientific heritage is a complex task as it requires a paradigm shift to fully understand the value of cultural heritage (Muñoz Viñas, 2020).

The available evidence suggests that there has been a shift in the way academic institutions approach their heritage and the importance of conserving cultural values. In terms of scientific advancement, outmoded instruments, methods, and practices are being superseded by new ones. At some stage of scientific progress, preservation and grouping of elements ceased to entail rejection of innovation. Instead, it denotes an adherence to procedures and intangible value, and a desire to rejuvenate it in the present. This phenomenon is particularly evident in university collections and museums, as exemplified by Soubiran (2008).

How can we preserve the intangible heritage? Intangible heritage encompasses values that determine a broader and contemporary conception of culture, including the ways of life, social practices, knowledge, skills, and mentalities of various individuals and groups within a community (Avrami et al., 2000). Academic knowledge is thus preserved in its immaterial form (Table 1).

**Table 1:** Elements of intangible scientific heritage.

Scientific heritage	Hard sciences	Biography Scientific work Sites of memory
	Soft sciences	Epistolary heritage Identity Methodological heritage Advocacy, social, and political engagement

From a normative safeguarding perspective, legal experts have emphasized the importance of categorizing all cultural heritage as intangible heritage to effectively safeguard it and understand its interconnections, while also maintaining flexibility and adapting adequate techniques for each type of heritage, whether tangible or intangible (Vaquer Caballería, 2005, p. 98).

It is important to note that standards alone do not encompass the full conceptualization of safeguarding cultural heritage. Protection of cultural heritage involves a lengthy process of identification and recognition, beginning with UNESCO’s traditional taxonomies. The academic framework offers compelling insights and evaluations of established concepts, including considerations of their boundaries, the potential for various events or activities to qualify as heritage, and the responsible parties for safeguarding heritage legal rights (Castillo Ruiz, 2022). On the contrary, when considering the epistemology of preserving heritage, the concept of cultural heritage as we comprehend it in the 21st century would not exist without the mechanisms of asset registration and identification in their respective countries of origin, alongside political and administrative recognition.^8^ The legal field considers the concept of heritage, as well as its protection and the role of the conservator-restorer (Alegre Ávila, 2018, p. 407; Rotaeche González de Urbieta, 2021). Furthermore, those working in the field have typically acquired skills and developed critical thinking while studying. Other studies examining the safeguarding of intangible heritage, particularly in relation to the Italian case, emphasize the development of the legal framework surrounding the protection of cultural heritage or the Spanish research on intangible heritage (De Giorgi Cezzi, 2009; Carballeira Rivera, 2021).

In the process of legal preservation, including new heritage (ranging from objects to living processes) that has taken place in recent decades, there has been a shift from solely relying on aesthetic or historical values to also considering identity, cultural value, and the ability of heritage to engage with collective memory in the present (Martínez Pino, 2012). This facet is undoubtedly reflected in intangible scientific heritage, resulting from creative activities that interact and increase knowledge (social, cultural, and heritage-related). Intangible aspects of a legacy, frequently oral, can aid in the acceptance of cultural diversity as a source of enrichment for humanity as a whole. Moreover, it highlights the idea of preserving the ‘spirit’ rather than the ‘material,’ aligning with Oriental philosophical thought (Vecco, 2010, p. 321).

**Figure 1:**
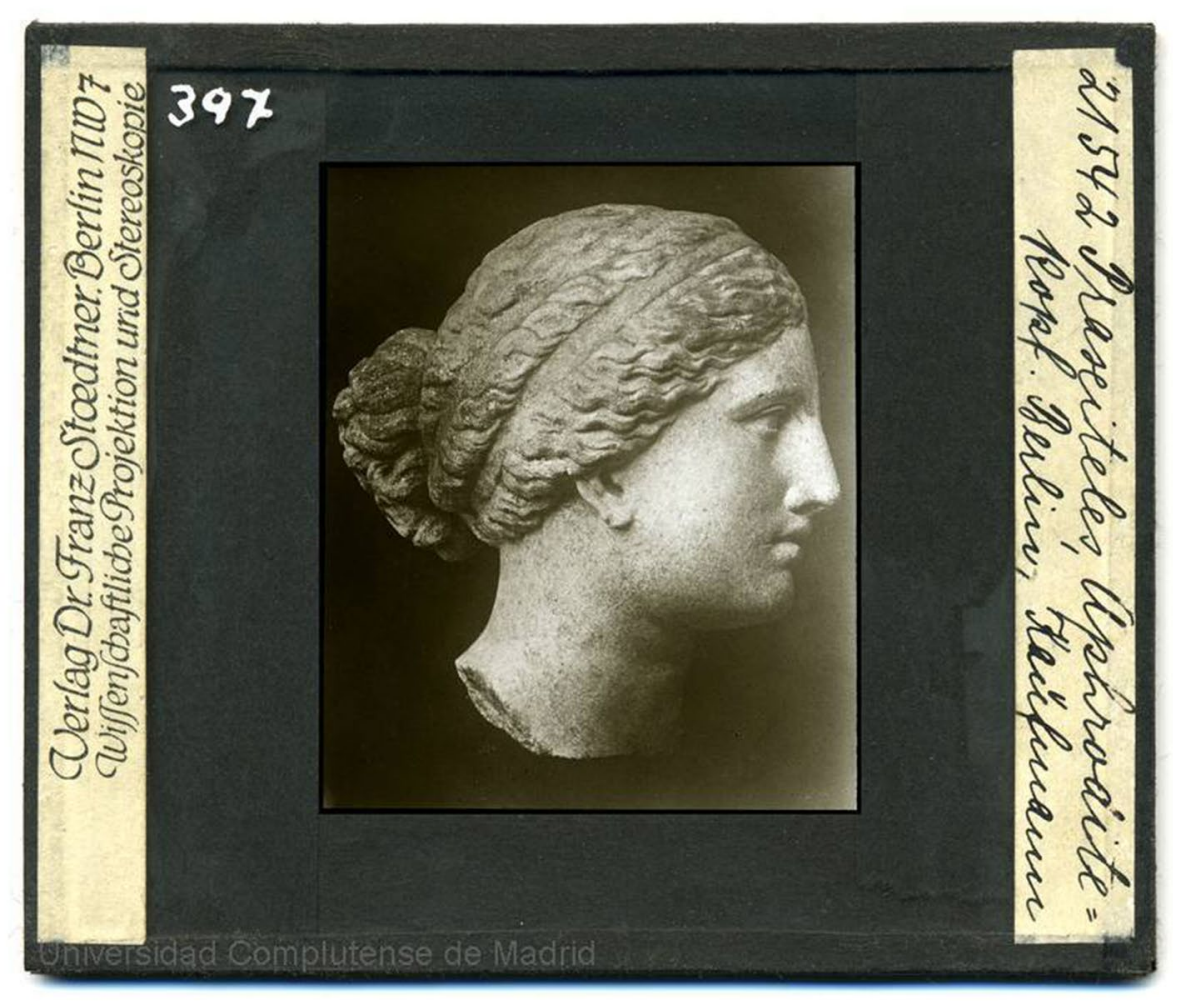
Archives Lafuente Ferrari. Praseiteles Aphrodite Kopf. Berlin, Kaûfmann. (Stoedtner, Franz, 1870–1944.). Complutense Digital Heritage, Universidad Complutense de Madrid. Reprinted from Complutense Digital Heritage by Universidad Complutense de Madrid, licensed under CC BY 4.0.

Scientific activity represents a compendium of knowledge that contributes to the progress of civilization through different forms of communication or access. These manifestations can take tangible forms or exist as a series of intangible expressions, among which artistic forms serve as a means of expressing research in a symbiotic relationship between art and science (Figure 1).

### Some experiences about the intersection between conservation and communication and the interplay between science and art

In the pursuit of critical work, there has been a paradigm shift in conservation practice. Ethical and theoretical conditioning factors, alongside social considerations, shape conservation-restoration practices (Marçal, 2023, p. 131; Marçal, 2021).

Additionally, we now emphasize the societal impact of heritage. A crucial aspect is to exhibit and convey the significance of heritage, illustrating how it can influence the standard of living, foster progress in our society, and yield tangible advantages while enhancing educational skills and personal development (Magar, 2023). However, in a world increasingly complicated by systemic inequalities and conflicting value systems, conservation’s ability to promote diverse forms of transmission remains uncertain. In this context, the transmission of heritage is vital for its preservation; without it, ethical conservation cannot materialize, and the sharing of its values becomes crucial (Belishki and Corr, 2019).

Artistic expression has the potential to serve as a means of communicating scientific ideas across all scientific disciplines, enriching the current body of knowledge and acting as a tangible or intangible legacy, as we will demonstrate below.

An instance showcasing the interplay between the scientific history of different fields and its artistic representation is the ‘Art and Science of the 21st Century’ exhibition, which took place in 2021 in Madrid. It was organized by the Spanish National Museum of Natural Sciences-CSIC in commemoration of its 250th anniversary alongside the Arcilla Foundation. The exhibition had a catalog listing and aimed to cultivate scientific awareness in society through art (Cánovas Fernández, 2021).

**Figure 2:**
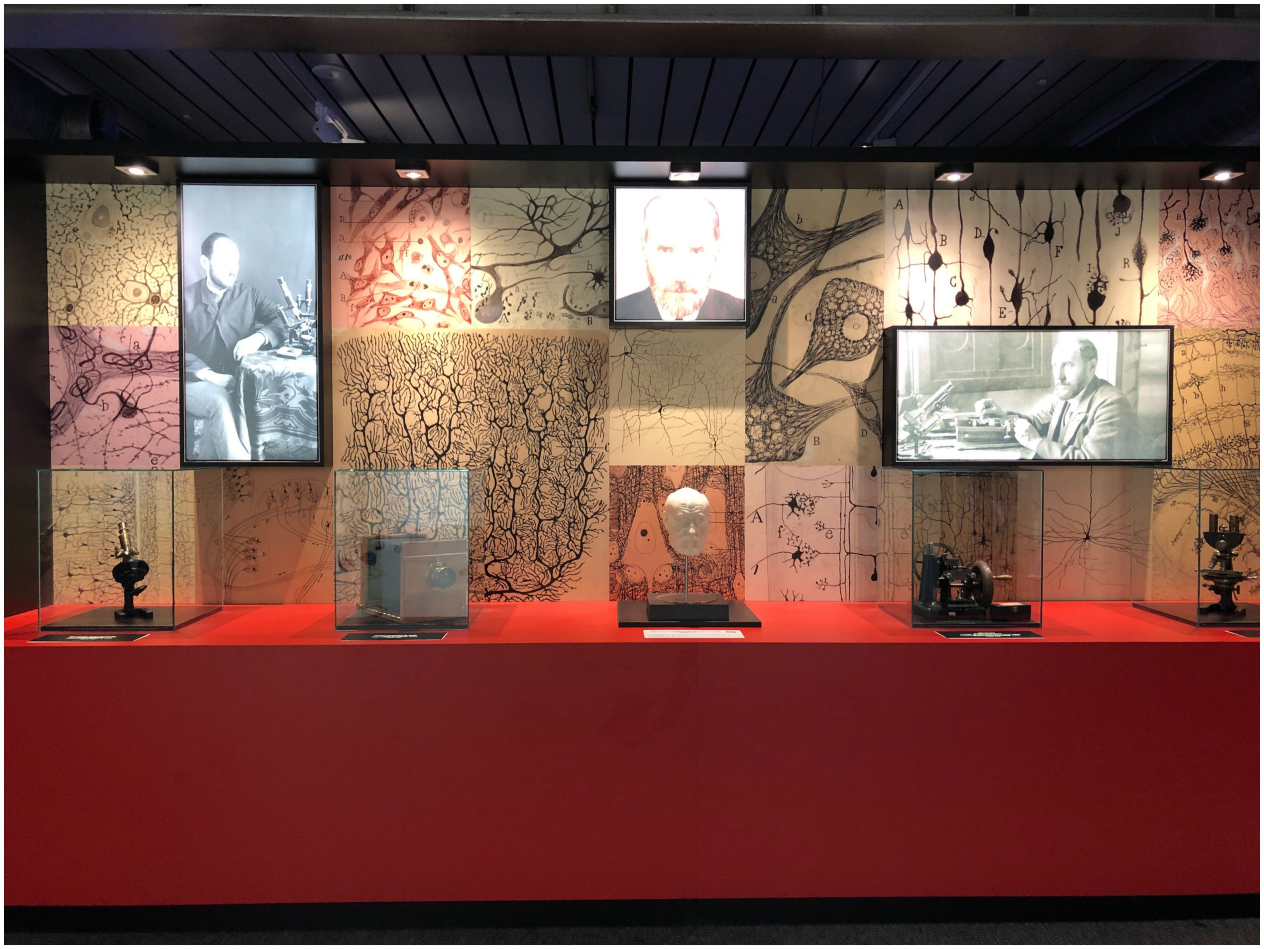
Photograph of the Exhibition “Santiago Ramón y Cajal” at the National Museum of Natural Sciences in Madrid (Spain). Note: Photographed by the author.

**Figure 3:**
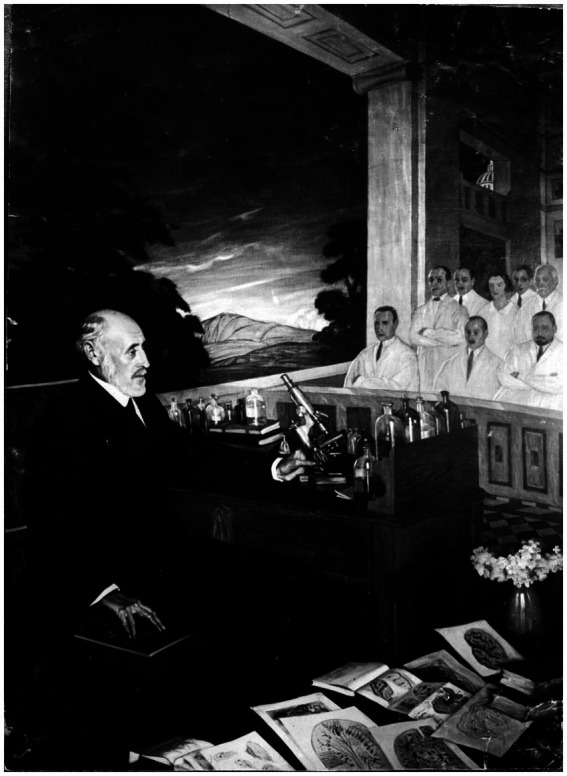
Painting of Santiago Ramón y Cajal in a master class with drawings and a microscope. By Ramón de Zubiaurre (1882–1969). Moreno House. Archive of Spanish Art (1893–1953). António Passaporte. Archivo Loty, IPCE, Ministerio de Cultura y Deporte. Licensed under CC BY-NC-ND 4.0.

The Ramón y Cajal Legacy, winner of the Nobel Prize in Physiology and Medicine in 1906, is managed and protected by the Spanish National Research Council (CSIC) as part of the scientific and personal legacy of the researcher. The Museum of Natural Sciences hosts a semi-permanent exhibition that includes scientific instruments, as well as intangible values of a humanist and artistic essence. Accessible in exhibition format, this legacy has achieved World Heritage status since 2017 (Figures 2, 3).^9^

In the context of academic discourse, with a slight foray into the artistic vanguard, we will examine the performative actions of artist Joseph Beuys (Cercós i Raichs, 2017; Hinrichs and Sartorius, 2021). As a member of the Fluxus group and an academic, Beuys believed that his concept of art encompassed not only the political realm but also empowered individuals to shape both their environment and themselves^10^. This interdisciplinary perspective includes science, making art and science essential components of education.

Recognizing the critical role of art and science in education, we understand that they contribute to the creation of new knowledge and a deeper understanding of ourselves. Beuys’ artistic performances and academic lectures challenge traditional boundaries, emphasizing the potential and limitations of different languages. To fully grasp and define the value of his immaterial legacy, alternative perspectives and methodologies are necessary.

The photographic documentation of Beuys’ blackboards, used during academic performances, constitutes a significant material legacy. While these blackboards serve as vehicles for his performative acts, they also contribute to an intangible legacy—one that inspires countless academic and performative actions. Museums, such as the Tate Modern in London, display objects related to Beuys, but these artifacts alone may not fully convey his extensive artistic and research contributions or the depth of his intangible legacy.

**Figure 4:**
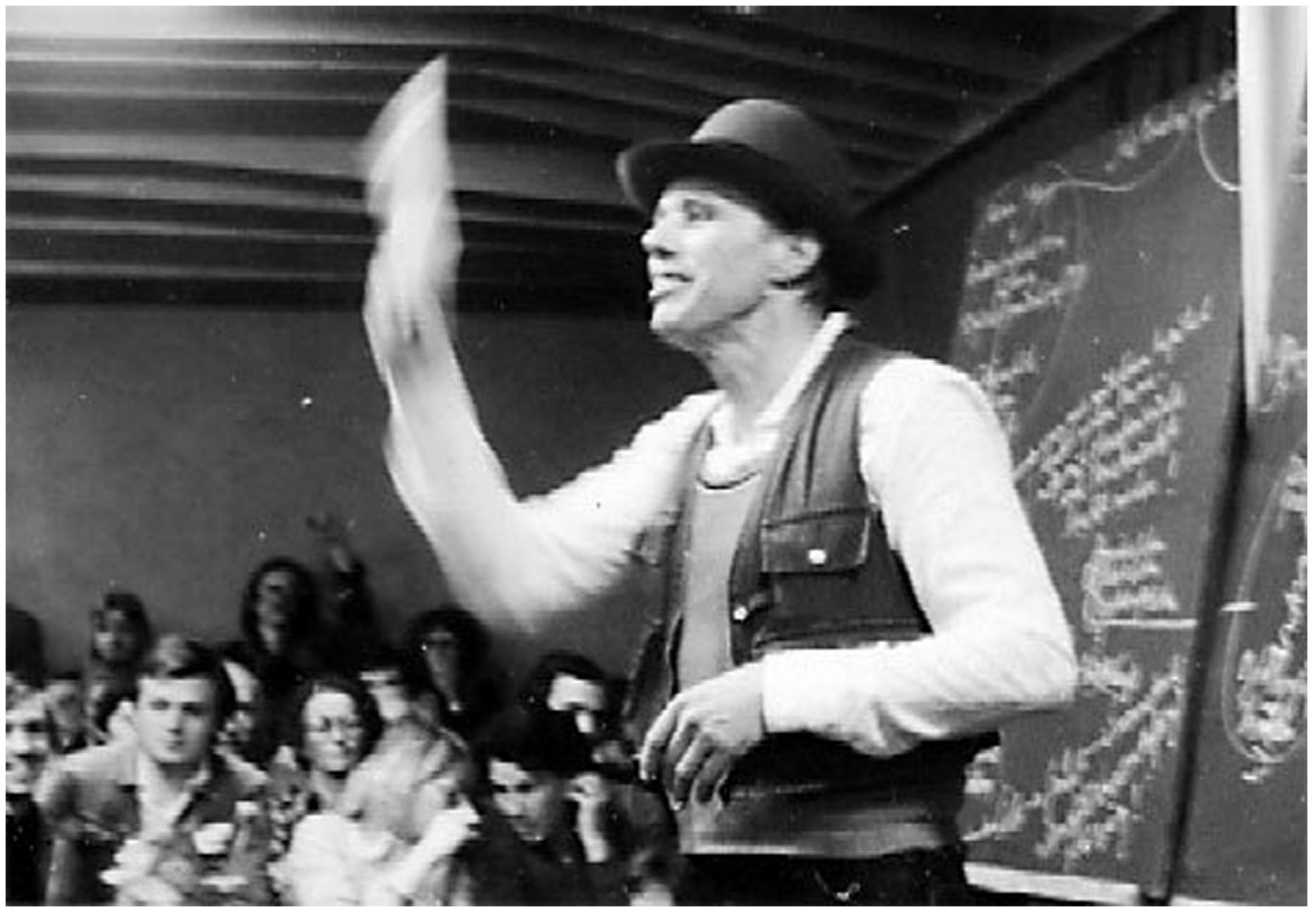
Image. Photograph. Joseph Beuys on his lecture “Jeder Mensch ein Künstler - Auf dem Weg zur Freiheitsgestalt des sozialen Organismus by Rainer Rappmann licensed under CC BY-SA 3.0 via Wikimedia Commons’’.

Preserving Beuys’ legacy, especially concerning the blackboards that served as both academic and performative tools, presents challenges. Originally intended as dynamic communication surfaces for writing and erasing content, blackboards were not designed for permanent records^11^ (Figure 4).

### Question: art or science?

Let us shift our perspective and begin by examining the activity of writing and publishing research results, which we understand as the activity of communicating scientific heritage, from another point of view. We’ll explore the relationship between art and science and other related aspects. Our goal is to establish a theoretical framework that encompasses what we have observed so far, within the broader context of a theory of society. This theory serves as both a tool for theoretical analysis and the scope of our proposal. Furthermore, we’ll consider how new technologies, as we’ll discuss later, impact this contemporary perspective.

Michel Foucault’s exploration of authorship during the transition from early modernity to its mature phase is thought-provoking. He poses the question: What is an author? (Foucault, 1969). This inquiry leads us into complex territory, touching upon the historical evolution of the concept of authorship.

In the context of modernity, the relationship between an author and their work undergoes significant shifts. Rather than focusing solely on the concrete individual, Foucault emphasizes the epistemic function—the role an author plays in shaping knowledge. The author’s name becomes a crucial reference point, allowing for classification and inclusion or exclusion of texts within specific corpuses. This attribution of artistic or cognitive value then determines a work’s social acceptability.

Foucault challenges the notion that an author is merely the owner of thoughts, ideas, words, and style within their work. Instead, he situates the author within the broader episteme of their era. This perspective rejects the idea of attributing an idea exclusively to a single author. Consequently, Foucault suggests a process of deindividualization in communication—a theme echoed by Roland Barthes (1977). However, Foucault takes this process further, emphasizing the distinction between scientific and literary discourse—the concept of the ‘author-function.’ In scientific discourse, where knowledge is systematically organized, the author-function is unnecessary. The validity of scientific statements relies on demonstrable truth, not the author’s identity. In contrast, literary works depend on authorship for recognition, tied to the author’s name (Longo and Magnolo, 2009).

Foucault’s discourse intrigues us due to its exploration of the art-science divide. We can extend Foucault’s approach to address recent developments in scientific publishing (our intangible heritage) and information technologies (ICTs). To do this, we turn to Niklas Luhmann’s systems theory. According to Luhmann (2005a), modern science and art represent two functionally differentiated systems. In scientific research, an abstract concept of truth suffices—knowing who said something is secondary to validity. Scientific truth transcends social, temporal, and spatial dimensions. Yet, the author’s role persists. Foucault acknowledges its importance, albeit unrelated to scientific validity. Luhmann, in turn, highlights reputation (akin to Foucault’s author-function) as essential for organizing the scientific system. Reputation enables selection from the vast pool of scientific information and publications (Luhmann, 2005a, pp. 291–316, 1998, pp. 244–51).

In this paragraph, we delve into the issue of scientific publications, considering them as part of our proposal on intangible heritage. We examine scientific communication from the perspective of humanities and social sciences. Rudolph Stichweh (2006) distinguishes between natural sciences (which primarily use journal articles), humanities (often favoring books), and social sciences (occupying a middle ground). It is quite obvious how, in the scientific system, the author’s name plays its role for a reputation-based self-organization of the system. Stichweh notes a standardization of scientific communication achieved through formal requirements like citations, which construct references to other authors and contribute to reputation (Stichweh, 2006). The Internet has revolutionized academic publishing, particularly through Open Access. Institutions across Europe adhere to the Berlin Declaration on Open Access to Knowledge in the Sciences and Humanities. This recognizes the Internet’s fundamental role in distributing scientific knowledge and cultural heritage. For the first time, we can create a global, interactive representation of human knowledge, ensuring worldwide access. The Berlin Declaration addresses the challenges posed by the Internet, aiming to modify scientific publishing and quality assurance systems. It aligns with the spirit of initiatives like the Budapest Open Access Initiative, the ECHO Charter, and the Bethesda Statement on Open Access Publishing.

As we continue our exploration, we’ll consider how these insights intersect with contemporary technological advancements.

### Answer: Parallelpoesie

We answer here the question opened with the previous paragraph: art or science? To do so we resort to a statement by Luhmann that has had much resonance in the Arts, but not instead, as is often the case with this author, in the sociological sphere.

We draw upon a well-known quote from Niklas Luhmann in the field of artistic research. By doing so, we not only benefit from the English translation of Luhmann’s words but also consider the perspective of this research domain. This perspective, in some ways, complements sociological theory and aligns with our adopted viewpoint and proposal. Let us quote directly from Lucia Ruprecht’s text (2019, pp. 174–175), referencing Luhmann’s statement:

“Monika Rinck quotes Niklas Luhmann, who points out that scholarship is not lacking in “gelehrter Prosa” (learned prose), but in “gelehrter Poesie” (learned poetry), which, according to Luhmann, is the only mode that is able to give expression to the “eigentümlichen Weltstimmungsgehalt wissenschaftlicher Theorien” (specific mood value of academic theory). Theory, Luhmann suggests, should always be accompanied by “eine[r] Art Parallelpoesie… die alles noch einmal anders sagt und damit die Wissenschaftssprache in die Grenzen ihres Funktionssystems zurückweist” (a kind of parallel poetry which says everything that theory says in different words, thereby limiting theoretical language to the confines within which it is functional).”^12^

In the realm of social sciences, we encounter the same Luhmann quote. This reference allows us to further explore the distinction between science and art, adding more insights. Taking a sociological perspective, we revisit the concept of Weltstimmungsgehalt (specific mood value) mentioned in the context of artistic research. German sociologist Koller (2019, p. 267) refers to the original text, where Luhmann distances himself not only from the political interpretation of his theory but also from its application by, let us translate directly from German, “pedagogues, historians, theologians, jurists, and philosophers.” This refers to Luhmann’s (2008, p. 199) discussion of the Zwischenthema (intermediate topic) in his essay *Unverständliche Wissenschaft*, addressing the language of science, particularly sociology. Luhmann concludes with the quote we have seen: “Soziologie ist eine export-intensive Wissenschaft geworden” (sociology has become a strong export discipline). Perhaps this explains the expectation that sociology should be highly comprehensible, given its role in interdisciplinary discourse. Interestingly, Luhmann views the unintelligibility of his theory positively—it guards against overly rapid appropriation, using intentionally a business jargon, by politics, pedagogy, history, law, and philosophy.

He then asks whether this indicates that sociology is monopolizing control over the definition of social reality. His answer is nuanced, but we’ll focus here on what might appear as a diminishing of sociology’s importance (and perhaps that of the social sciences and science in general). According to Luhmann (2008, p. 199), ‘It cannot be claimed in any way that sociology, as a science, currently adequately explains social reality. It possesses no knowledge guaranteed to be true about our society. Therefore, its concepts and statements should not be uncritically accepted as knowledge in other disciplines.’

However, Koller (2019) suggests that Luhmann’s stance opposes a ‘scientific narrative.’ Luhmann’s ‘gelehrte Poesie’ asserts that the language of science should resemble the language of art, bridging two seemingly disparate systems. This intriguing statement relates both to the topic explored in this article and to the broader intersection of science and art. Sociologist Koller, along with authors like Roberts (1999) and Schwanitz (1999) from literature, philosopher Balke (1999), and artist Bunsen (1999), views Luhmann’s theory as a form of art—a theoretical construction that transcends disciplinary boundaries (Koller, 2019, p. 264). It’s essential to clarify that we are discussing the language of theory here, with Luhmann emphasizing not only words but also ‘contexts of selection’ (Luhmann, 2008).

There are various quotes discussing Luhmann’s writing style and how he constructed his theory. These quotes highlight its artistic traits, and Luhmann (1987a, 2008) and Koller (2019) himself acknowledges this when discussing theory and scientific writing. Let us revisit one point here to conclude the paragraph and consider its potential development in relation to our theme. A second point will be introduced later.

The first point brings us back to the challenge of political interpretations of theory (and external interpretations in general), leading us to Luhmann’s quote about Weltstimmungsgehalt (world mood content) and the role of Parallelpoesie (parallel poetry). Luhmann (2008, p. 200) clarifies that the issue in theory formulation is not merely the difficulty of understanding the theory’s language. Instead, he poetically states: “Only a brief window of time exists for the listener’s or reader’s linguistic attention; only a small span in order to accommodate words, thoughts, and associations. Then one must release control and rely on the partner’s memory. But how can we predict or influence what the partner reactivates at any given moment? How do we prevent unrelated conceptual traditions or biases from constantly resurfacing? All of this demands significant text compression. Sometimes word economy helps, but then we face the challenge that readers read too quickly while listeners hear too slowly. Thus, sentence structure must be both smooth and elegant, surprising yet familiar, capturing attention and aligning with the style of the theoretical statement. I previously mentioned simultaneous presence—that’s the crux of the matter.”

While comprehensibility cannot prevent us from expressing what is possible to say, Luhmann concludes that political interpretation does not necessarily provide a second satisfactory version of the true content of a theory. In such cases, we must instead rely on what we have termed ‘Parallelpoesie,’ which can reintegrate scientific language within the boundaries of its system (Luhmann, 2008, pp. 200–201).

As we continue our exploration, we’ll consider how these insights intersect with contemporary technological advancements and their consequences on our issue. We leave aside Luhmann’s proposal with regard to the relationship between art and science, language, and (sociological) theory. Instead, we focus on some consequences of new technologies on scientific publications and the organization of knowledge in general. From here, following the suggestions of the Berlin Declaration that we have anticipated and that reinforce our hypothesis in relation to the intangible heritage of the humanities and social sciences, we should resume and point out the reference to the Digital Humanities, before observing, among some examples, the one in relation to which we intend to formulate our proposal.

### New technologies and their impact on science communication

What is happening to scientific communication with the development of new media, and the Internet in particular? In this section we are interested in dealing with new communication technologies with a view to their application to the issue we are dealing with and what we have somewhat only foreshadowed with the reference to the Digital Humanities. A development, the latter, which in some ways seems to have bridged the gap between Science and the Humanities. But let us take a closer look at this issue before drawing the final conclusions.

We could say, using an expression from Niklas Luhmann, that everything begins with having already begun (Alles beginnt mit dem Schon-begonnen-haben). The field of Digital Humanities had already emerged by the 1970s. Even then, a cyberneticist—whom we will quote several times—expressed concern about the contrast between the soft sciences and the hard sciences. While the hard sciences grappled with soft problems, the soft sciences faced the challenge of measuring themselves against hard problems. von Foerster (1972), the cybernetician in question, hoped that the hard sciences (including cybernetics) could contribute their expertise to solving the hard problems of the soft sciences. However, he did not discuss applying the methods of the former directly to the latter’s research. The reductionist approach of breaking down objects into progressively smaller parts, which von Foerster refers to, would not succeed with the subjects of inquiry in the soft sciences: “My suggestion is that we apply the competences gained in the hard sciences—and not the method of reduction—to the solution of the hard problems in the soft sciences. I hasten to add that this suggestion is not new at all. In fact, I submit that it is precisely Cybernetics that interfaces hard competence with the hard problems of the soft sciences” (von Foerster 1972, p. 192).

With this suggestion, von Foerster acknowledged the complexity of the systems that constitute the objects of the soft sciences (such as psyche, society, culture, and language). In these systems, studying a single part is inconclusive regarding the functioning of the whole; instead, their functioning emerges from interactions among their parts. Closer to our current context, von Foerster argues that the expertise of Cybernetics should be measured against the problems faced by the soft sciences.

One such problem, particularly relevant to society, is the participatory crisis that excludes individuals from active participation in the social process. von Foerster attributes this crisis to the development of traditional mass media, which enable one-to-many communication but lack a response channel, rendering interaction between individuals and society impossible. While this analysis is not new (Thompson, 1995), what strikes us is the framing of the issue from a cybernetics perspective and the prophetic anticipation of later developments facilitated by new technologies—specifically, computers and the Internet. Indeed, these technologies seem precisely to have intervened to solve the problem of the lack of interaction between the individual and society, enabling many-to-many communication in the contemporary world, transcending geographical restrictions (Longo, 2005). “Traditional mass media have been based on one-to-many communication. Hence the Internet has a large intrinsic democratic potential. In the terminology of Vilém Flusser it can be said that it could support a shift from discursive media society to dialogic media society” (Fuchs, 2005).

But it is not only about this. That of technological impact is a widely studied and debated issue, and the scenarios arising from this development have been variously described (Castells, 1996, 2001a,b), while bursting in to complicate the picture, transcending even more established distinctions, is artificial intelligence (Magnolo and Taurino, 2018; Magnolo and Pellerino, 2020). To return to the consequences of the development of new media that interest us most closely, there would be innovative ways of organizing knowledge and society, through the production of knowledge no longer the result of academic research alone, but also resulting from the interaction in cyberspace of ideas, information and new mental schemes (Levy, 1997). Alongside what has been called the hyper generalization of online communication, namely its separation from the contexts in which it was produced (Esposito, 1995), there then emerges, only seemingly in contrast to this, a personalized selection and use of information in relation to the user’s interests (Landow, 1992).^13^

Let us attempt to collect the suggestions we have reported before moving on with our discussion. On the one hand, to take up the issue of the importance of the author as a selection criterion within the scholarly system, author-function seems to have lost its relevance precisely because of the high level of personalization in the use of the Web. At the same time, the differentiation of increasingly specialized disciplines, scholarly organizations and journals, distributing and coordinating information, which had characterized the differentiation of the scholarly system and its internal organization, are undermined precisely by the digital formats that now replace printed publications and can circulate on the Internet in OA format (Vanderstraeten, 2010).

von Foerster in this context somewhat anticipated the use of search engines and even artificial intelligence itself, in the sense that he was already talking about network interaction and machine learning, where it is the search itself that helps to produce through its unfolding one of many catalogs of knowledge. A scenario that corresponds to what Levy (1997) has called Cosmopedia. Rather than on the question of the author, we could say that the question of the organization of knowledge and the selection of information is played out in relation to the overcoming, by telematic media, of the printed book, as an expression of analog media linked to that medium, to the idea of library and catalog in the traditional sense. To some extent, what the Berlin Declaration hoped for has already been realized: with the digitization of content and its consequent dissemination on the Internet, the limitation represented by the book has been overcome with regard to “linearity and consequentiality in the arrangement of themes and information and that very rarely as in the case of encyclopedias can be interrogated more loosely allowing a combinatorial freedom that encourages the reader’s linking abilities” (Cevolini, 2008, p. 75).

Let us summarize the point we are interested in here to conclude the paragraph:

- Thanks to new digital technologies and the Internet, paper publication is not the only mode of communicating the results of scientific research;
- As far as the Humanities and Social Sciences are concerned, the digitization of publications and their availability through the Internet on repositories, digital platforms, etc., according to the principles of the Berlin Declaration, reinforces the consideration of this body of knowledge as (intangible) scientific heritage;
- From the perspective of the heritage disciplines this form of “publication” would fulfill at the same time the function of the preservation of this heritage and its communication or enhancement;
- This technological development challenges the relevance of the author as a criterion of information selection and knowledge organization (hyper generalization of communication and personalization of content selection);
- At the same time, the book and traditional libraries also lose relevance with respect to the possibility of processing and storing knowledge in a kind of memory, where information is no longer, therefore, presented in a linear and ordered form, but discontinuous and disaggregated, and it is the reader who constructs his or her own reference document;
- This “container” of knowledge does not need to be ordered like traditional libraries, nor therefore to have a catalog that reproduces that order and allows one to find the information, but to build links between the information within it: “This would then allow for cross-reading that not only disregards authors but also disregards disciplines” (Cevolini, 2008, p. 77; von Foerster, 2008).

### Digitization as preservation of scientific intangible heritage

We have been attempting to establish a legitimate path for considering scientific production in the Social Sciences and Humanities as intangible cultural (scientific) heritage. This reflection draws on heritage disciplines, and sociological theory has allowed us to bridge the gap between communication in the hard sciences, the soft sciences, and the language of art. Now, we need to revisit how we communicate science, focusing on preserving and enhancing our intangible (scientific) cultural heritage. Sociological theory provides a concrete reference point for this endeavor (Buchinger, 2012).

Simultaneously, we have reconsidered Digital Humanities as an evolution of hard science expertise applied to solving complex problems in the soft sciences. From this perspective, we face the possibility—already widely used—of organizing, accessing and communicating available knowledge differently than in the past. The Internet and digital formats serve as containers for this freely accessible and consultable intangible heritage. However, they also present challenges in terms of misrepresentation and societal value of this heritage.

A proposal suggests utilizing Digital Humanities as a procedural framework for conservation, building upon critical conservation practices and incorporating current developments in conservation education.

The integration of conversation-conservation^14^ processes would facilitate multidisciplinary knowledge exchange and shift decision-making toward a more comprehensive approach (Fekrsanati and Schimmeroth, 2023). It is acknowledged that an interplay exists between communication, decision-making, and actions in the preservation of cultural heritage (Marçal and Gordon, 2023).

Starting from communication, as we have done in our work with the use of terms, we recognize the difficulty of setting limits regarding material and immaterial aspects. Through our examination of language (terms and concepts), we explore methods for restoring, communicating, transmitting, and experimenting with the intangible legacy. Conservation is discussed through written, oral, and visual language in both academic and artistic fields, reflecting the duality of art and science. Researchers also face the challenge of incorporating virtuality within academic heritage, enhancing its transmissibility in the university environment. The crucial role of communication lies in developing digital strategies that position this valuable (also intangible) heritage, focusing on two key areas: the emerging role of information and communication technologies in museums and university collections, and promoting experiences that catalyze positive changes in associated digital practices (Vanrell Vellosillo, 2019). While an exhaustive study would be necessary, we present some interesting examples of digitization of academic, tangible, and intangible heritage. One of them is particularly important for our discussion.

On one hand, the collaboration between the Complutense University of Madrid’s project, PDC (Patrimonio Digital Complutense), and networks such as EUROPEANA and HathiTrust serves as a repository for preserving and providing access to the university’s heritage^15^. Specifically, the platform is easily accessible to the university community, especially those focused on conservation-restoration, either directly or through virtual exhibitions. Through initiatives like the Cabinet^16^, the library and student communities collaborate to discuss and implement the knowledge presented on this platform, utilizing the digitized heritage (Lankes et al., 2007).

Developed by the Hebrew University of Jerusalem, the physicist “Albert Einstein Archives” website is significant in understanding the person of a Nobel Prize-winning researcher who transformed our understanding of the world from both experimental and humanistic perspectives^17^.

We draw special attention to the “Niklas Luhmann-Archiv,” a portal that serves as the telematics interface for the project “Niklas Luhmann – Theorie als Passion: Wissenschaftliche Erschließung und Edition des Nachlasses” (Niklas Luhmann – A Passion for Theory: Academic Indexing and Editing of the Legacy). This project focuses on the German Niklas Luhmann (1927-1998), who taught and conducted research at the University of Bielefeld from 1968 to 1993. Collaborating on this initiative are the Faculty of Sociology, the Archives and Library of Bielefeld University, and the Digital Humanities department at Bergisch University of Wuppertal. Their joint efforts aim to revive Luhmann’s heritage and enhance the visibility of his social system theory beyond his written works.^18^

The project is particularly interesting because it involves, and this is one of the reasons for our choice, archiving and digitizing Luhmann’s scientific legacy and making it available on an *ad hoc* portal. This is similar to other initiatives where we build containers that preserve and make available knowledge deemed worthy of being disseminated and passed on as scientific (cultural) heritage. But that is not all that is involved here. The perhaps central part of the project is the use of Luhmann’s Zettelkasten (file cabinet), which gives unity to Luhmann’s intellectual work, adding further elements to our general thesis and to the case at hand from the point of view of its recognition as (intangible) cultural heritage:

“Niklas Luhmann’s extensive scientific legacy makes the author and his theory visible beyond his published works. This applies in particular to the actual center of Luhmann’s theoretical work: his file cabinet. The notes, presumably written between 1952 and the beginning of 1997, with the help of which Luhmann systematically organized the results of his extensive and interdisciplinary reading, document the development of theory in a unique way, so that the collection can also be understood as an intellectual autobiography. In addition, the card index has a specific organizational structure that not only made it the indispensable theory development and publication machine for Luhmann, but also makes it interesting in terms of the history of science.”^19^

All these formulas for the identification, preservation, and cataloging of cultural heritage are necessary to promote its accessibility and enhancement, and to give direction to the transfer of knowledge set out in this paper.

At this point, what would be the applicable formulas to promote accessibility and enhancement, taking digitized repositories as a starting point? Through critical museology and museography tools, previously documented knowledge can be interpreted and experienced, generating living knowledge and cultural memory. Academic museums have reflected on the design of policies for safeguarding material elements and intangible heritage, their management, and the ways in which they are made accessible to the public. They agree on the progress and changes in scientific institutions to make their heritage public, and the complex relationship of the scientific community with institutional heritage. In this way, it is necessary to transfer a scientific legacy that can continue to be interpreted and generate more knowledge, fostering an active relationship between legacy and society.

## Discussion

After analyzing scientific heritage, particularly intangible heritage, and proposing a path for its conservation through digital humanities, several key points emerge. The use of language and terminology, as well as the similarities between scientific heritage and work across all scientific disciplines—including mathematics, medicine, biology, geology, aeronautics, anthropology, history, ethnography, literature, politics, and philosophy—should be highlighted. The essays analyzed reflect an intangible value, albeit not explicitly stated. They explore aspects of memory values, identity, social activism, nation, epistolary heritage, and memory landscapes.

A double paradigm shift could be undertaken to approach the preservation of intangible scientific heritage: from viewing scientific heritage as solely technological (material and technical) to recognizing it as experiential (intangible) heritage, and moving toward critical conservation.

In this sense, the digital humanities offer a preservation dialog where tangibles and intangibles are identified and documented through digitization. This approach allows for knowledge acquisition and virtualization of metadata incorporating concepts, terms, and images via intermediary channels (libraries). These repositories act as containers for preservation awaiting utilization. Preservation involves the transfer of a scientific legacy that can continue to be interpreted and generate more knowledge, fostering an active relationship between legacy and society. This transfer takes place through communication in written, oral, and visual language and in various fields, including academia and art, which encompass both art and science. This transfer of scientific legacy enables it to be interpreted and generate additional knowledge, creating a symbiotic link between legacy and society.

It is necessary to examine applicable formulas for experimenting with digitized repositories as a starting point. As a conclusion and future extension of these theoretical reflections on preserving scientific heritage without physical material, we propose taking a step toward enhancing their value through critical museology and museography. Using critical museology and museography tools, previously documented knowledge can be interpreted and experienced, resulting in a “living” knowledge and cultural memory that can be integrated into current intangible heritage taxonomies and protected by legal frameworks, offering complete safeguarding guarantees.

A further step should be taken in the case of the Luhmann Archiv. Continuing to use concepts from the heritage disciplines and with reference to the development of the activity of museums, it is a matter of moving from that historical phase in which museums acted as containers to the contemporary phase in which museums open up to converse with communities. This parallelism highlights how the activity of preservation and enhancement allows us to apply the conceptuality proper to institutions traditionally in charge of cultural (tangible) heritage, such as museums, to digital platforms or repositories, which archive, preserve, and enhance intangible (scientific) heritage. For the latter, too, it is a matter of going beyond the container stage. The new technologies that make these repositories possible also offer a variety of tools useful for this purpose. The use of the Zettelkasten as a guide, as a proposal for cross-reading and transmitting the value of Niklas Luhmann’s legacy, is a step in this direction. His theory is very complex, and the completeness of the material collected and made accessible is not in itself a guarantee of understanding his thought, if we are to interpret the value stakes of his legacy in this sense (Bucchi, 2013; Bucchi and Trench, 2021). Therefore, it is necessary and useful to have tools to facilitate its understanding through the provision of glossaries of concepts and the illustration of cross-references between them.

Luhmann’s systems theory, as a whole, is a theory of society, the value of which lies in a description of modern and contemporary society that everyone, as a citizen or researcher, could use to understand the mechanisms of central institutions of our society such as democracy, constitution, human rights, and equality, among others. At the same time, it is a theory somewhat formalized by virtue of its conceptual apparatus, necessitating tools to facilitate its understanding.

If we take up the analysis conducted on Luhmann’s scientific production with regard to art and science, it is pointed out (Koller, 2019, 269) how Luhmann’s own representation of his writing process is the same as his description of the process of artistic production. And when, in this regard, the question is raised as to whether Luhmann is then a scholar or an artist, Luhmann’s response in an interview (Luhmann, 1987b, p. 142) to the request to reveal the secret of his statement regarding the fact that his books write themselves is reported, which begins as follows: “Ich denke ja nicht alles allein, sondern das geschieht weitgehend im Zettelkasten. […] Meine Produktivität ist im wesentlichen aus dem Zettelkasten-System zu erklären” [I do not do all the thinking on my own, it’s largely done in the file cabinet. […] My productivity can essentially be explained by the file cabinet system]. He thus traces in the Zettelkasten an indispensable tool for his writing process. Without going into the details of the organization of the file cabinet that are explained, we can conclude as follows (Koller, 2019, p. 271): “Kein Wunder also kann Luhmann sagen: ‘Die Theorie schreibt sich […] selbst’. Ein kleiner Schritt wäre es dann zu sagen: Luhmanns Texte sind sich selbst programmierende Kunstwerke. Die Selbstreferentialität des für Luhmanns wissenschaftliche Produktion wesentlich mitverantwortlichen Zettelkastens besteht darin, dass jeder Zettel nur ein Element ist, ‘das seine Qualität erst aus dem Netz der Verweisungen und Rückverweisungen im System erhält’” [“No wonder Luhmann can say: ‘Theory writes itself […]’. It would then be a small step to say: Luhmann’s texts are self-programming works of art. The self-referentiality of Luhmann’s file cabinet, for which it is largely responsible for his scientific production, consists in the fact that each card is only one element ‘that only receives its quality from the network of references and back-references in the system’ “]. So, his response highlights the role of the Zettelkasten in his productivity. This tool is indispensable for his writing process, illustrating the self-referentiality of his work.^20^

To conclude, as Luhmann notes regarding theory, language involves not only words but also the transmission of ‘contexts of selection.’ While we inevitably rely on established concepts, scientific progress occurs when we question the existing framework rather than merely reproducing it. This leads to a challenging question: Should we persist in using terminology whose meaning has evolved, or should we abandon it to avoid losing connections with tradition? In the context of Luhmann’s theory as an ‘object of preservation,’ we must address the novelty—conceptual and otherwise—that this theory brings to the social sciences. Specifically, “how can we prevent unrelated conceptual traditions or biases from continually resurfacing?”

On the other hand, we have observed how the gap between hard and soft sciences has narrowed, thanks to processes envisioned by von Foerster and realized through Digital Humanities. This convergence is perhaps due, in part, to the prevalence of Open Access journals as the preferred mode of scholarly communication across disciplinary fields (Vanderstraeten, 2010). However, what stands out for our purposes is the breakdown of disciplinary boundaries within the ‘containers’ of knowledge facilitated by digital content on the Internet: this allows a cross-reading that not only disregards authors but also transcends traditional disciplines (Cevolini, 2008; von Foerster, 2008).

Considering this context, and recognizing that soft sciences might benefit from the language of art rather than that of the hard sciences, we pose a question related to the communication of Niklas Luhmann’s scientific legacy through digitization: What form of ‘Parallelpoesie’ could reintegrate scientific language within the boundaries of its system?

## Data Availability

The original contributions presented in the study are included in the article/supplementary material, further inquiries can be directed to the corresponding author.

## Footnotes

^1^‘The Declaration Of Halle. Academic Heritage and Universities. Responsibility and Public Access’ Universeum, European Academic Heritage Network (2000). https://www.universeum-network.eu/the-declaration-of-halle/ (accessed 2022-11-25).

^2^We support and extend to all disciplines the motto of Frontiers journal: Scientists empower society!

^3^Culture as a Good and as a Medium" Research Group, Department of Legal Studies, University of Salento.

^4^‘In fact, the university is seen as one of the most persistent and resilient of human organizations, reshaping and adapting as society changes and new challenges emerge’ (Economou et al., 2021).

^5^Research and analysis in the English language of the terms ‘scientific heritage of scientifics’ and ‘scientific heritage of social scientists’ with Google Scholar database search engine. Note: Thanks to the development of artificial intelligence, it is possible to translate texts into other languages and to access other ways of thinking and constructing research, especially in the field of cultural heritage. But, what technology facilitates is negatively counterbalanced and weighed down by anthropogenic factors such as war. Many of the sources on Eurasian research platforms that were consulted before the war in Ukraine in 2023 are currently inaccessible.

^6^UNESCO. ‘Dive into Intangible Cultural Heritage’ web-semantics and graphic visualization, proposes a broader conceptual and visual navigation through close to 500 elements inscribed on UNESCO’s Lists of the 2003 Convention. https://ich.unesco.org/en/dive.

^7^ICOM-UMAC, International Committee for University Museums and collections. ‘Worldwide database of University Museums and collections’. https://university-museums-and-collections.net/.

^8^A similar reflection can be made in the case of the historical trajectory of human rights: these are subjective rights whose possibility of being recognized depends on their inclusion in the legislation of states as fundamental rights within constitutions. Despite their supposed universality, they can only be recognized by national states (Ferrajoli, 2013).

^9^‘Archives of Santiago Ramón y Cajal and the Spanish Neurohistological School’. The scientific archives of the Cajal Legacy are included in the Census-Guide to the Archives of Spain and Latin America, since they were included in 2017 by UNESCO in the Memory of the World International Register of World Heritage. https://cajal.csic.es/el-legado-cajal/ (accessed 2022-11-25).

^10^Interview by Peter Holtfreter, Susanne Ebert, Manfred König y Eberhard Schweigert. Konununi1cation, n° 1, (1973) Düsseldorf, in DDOOSS website. https://ddooss.org/textos/entrevistas/entrevista-con-el-profesor-joseph-beuys (accessed 2024–107-29).

^11^Conservation of Joseph Beuys chalkboards by the State Museums Berlin, Hamburger Bahnhof. Staatliche Museen zu Berlin. https://www.smb.museum/en/museums-institutions/hamburger-bahnhof/collection-research/conservation-care/conservation-project-richtkraefte-directive-forcesjoseph-beuys-2013/ (accessed 2022-11-25).

^12^“The notion of parallel poetry exemplifies the specific achievements of artistic research such as the ‘researching art’ created and presented by contemporary dance and literature” (Ruprecht, 2019, p. 175).

^13^This begs the question, for example, whether the function of browsers can be understood as a form of control over access to information, or only as a tool that makes access to information available (Hargittai, 2007).

^14^Our reference for this choice of terminology is Cybernetics and Conversation Theory di Gordon Pask (1976).

^15^Complutense Library, ‘Complutense Digital Heritage/Patrimonio Digital Complutense‘, https://patrimoniodigital.ucm.es/s/patrimonio/page/exposiciones.

^16^Cybernetic archive for the preservation of cultural heritage: https://www.ucm.es/ficherocibernetico/.

^17^The Hebrew University of Jerusalem. Project Website ‘Albert Einstein Archives’. https://albert-einstein.huji.ac.il/about-us.

^18^Universitat Bielefeld. Project Website ‘Niklas Luhmann Archiv’: https://niklas-luhmann-archiv.de.

^19^ https://niklas-luhmann-archiv.de/nachlass/zettelkasten

^20^There are numerous scholarly articles in various disciplinary fields that have dealt with the system devised by Luhmann for the filing of texts. Regarding the socio-cultural field, please refer to Cevolini (2006, 2016) and Esposito (1996).
